# Differences in prevalence arising from reference values for physiological and laboratory measurements used in studies of Tibetan highlanders and differences in study populations between the two studies

**DOI:** 10.1186/s40101-026-00421-1

**Published:** 2026-02-04

**Authors:** Hiroaki Arima, Sweta Koirala, Takayuki Nishimura

**Affiliations:** 1 Department of International Health and Medical Anthropology, Institute of Tropical Medicine, Nagasaki University, 1-12-4 Sakamoto, Nagasaki, 852-8523 Japan; 2 Graduate School of Biomedical Sciences, Nagasaki University, 1-12-4 Sakamoto, Nagasaki, Japan; 3 School of Tropical Medicine and Global Health, Nagasaki University, 1-12-4 Sakamoto, Nagasaki, 852-8523 Japan; 4 Nepal Development Society, Ward 29, Naubise, Kaski District, Pokhara Metropolitan City, Nepal; 5 Department of Human Life Design and Science, Faculty of Design, Kyusyu University, 4-9-1 Shiobaru, Minami-Ku, Fukuoka, 815-8540 Japan

**Keywords:** Tibetan highlander, Nepal, Clinical criteria, Prevalence comparison

## Abstract

We conducted an epidemiological study of Tibetan highlanders in Tsarang Village, Mustang District, Nepal, and reported the findings in Arima et al. (Journal of Physiological Anthropology, 43:25, 2024). Subsequently, Sienna R. Craig and colleagues, who have conducted long-term research in the same region, published a commentary on our study, providing an opportunity for further scholarly discussion. In their commentary, Craig et al. highlighted differences between our study and theirs in the reference values used for disease classification, as well as the limited explanation provided in our article regarding the rationale for selecting these values. They further noted that these differences may have contributed to discrepancies in the summary and interpretation of population health indicators between the two studies. In this commentary, we seek to clarify the background and rationale underlying the reference values and analytical choices adopted in our study, and to discuss how differences in study design and population characteristics may influence prevalence estimates and interpretations of health status. Through this discussion, we aim to contribute to a more nuanced understanding of health assessment among Tibetan highlanders living at high altitude.

## Main text

### Perspectives on the health status of Tsarang residents

In their commentary, Sienna R. Craig and colleagues noted that our article could be interpreted as suggesting that the prevalence of chronic diseases among residents of Tsarang Village is high and that the population is therefore in poor health. They further advised caution in interpreting the prevalence of obesity, erythrocytosis, and low oxygen saturation among women in Tsarang, given that similar findings were not observed in their dataset.

We appreciate this opportunity to clarify our interpretation. In our original article, we described an increase in the prevalence of lifestyle-related diseases among Tibetan highlanders living in regions other than Tsarang, while noting that such increases were not clearly evident among current residents of Tsarang itself. Although we reported prevalence estimates based on the reference values adopted in our study, these findings were not intended to characterize the overall health status of Tsarang residents as poor or severe.

Rather, our discussion was informed by previous studies conducted in other Tibetan highland regions with different environmental and socioeconomic conditions, such as Ladakh and Qinghai Province, where increases in lifestyle-related diseases have been reported.

At the same time, we acknowledge that differences in disease definitions, reference values, and study design between our study and that of Craig et al. may reasonably lead to different interpretations of population health. We also recognize that the reference values adopted in our study, and the way they were described, may have conveyed impressions that were not fully aligned with our original intent. In the sections below, we therefore provide a more detailed explanation of the reference values used and discuss how these methodological differences may influence the interpretation of prevalence estimates.

### Interpretation of the prevalence of individual conditions

We begin by discussing the prevalence of obesity. In the commentary by Sienna R. Craig and colleagues, it is noted that our article reported that 26% of women were classified as obese, whereas no such cases were identified in their study. One possible explanation for this difference relates to terminology used in our article. We described residents with a body mass index (BMI) ≥ 25 kg/m^2^ as “obese” or as having “obesity,” whereas a more precise description would have been “overweight or obese.” When expressed in these terms, 25.6% of participants in our study were classified as overweight or obese. By comparison, Craig et al. reported that 13% of women in their dataset were overweight (BMI ≥ 25 and < 30 kg/m^2^), while none met the criterion for obesity (BMI ≥ 30 kg/m^2^). Upon reexamining our data in greater detail, we found that 20.0% of women were overweight and 5.6% were obese [1]. Accordingly, women meeting the criterion for obesity were present in the Tsarang population included in our study. Body weight in our survey was measured with participants barefoot, and 1 kg was subtracted to account for clothing. Although broadly comparable measurement procedures and classification criteria were applied in both studies, differences in prevalence were observed, which may reflect differences in the characteristics of the study populations.

We next consider the prevalence of hypertension. In our study, hypertension was observed in 17% of women, whereas Craig et al. reported a prevalence of 27%. We defined hypertension as a systolic blood pressure ≥ 140 mmHg, a diastolic blood pressure ≥ 90 mmHg, or current use of antihypertensive medication following a prior diagnosis [2]. Our study included participants aged 18 years and older, while Craig et al. restricted their analysis to women aged 39 years and older. This difference in age structure is likely to have contributed to the observed discrepancy in prevalence. In addition, under hypobaric hypoxic conditions, blood pressure—as well as hemoglobin concentration—may influence oxygen circulation dynamics. From this perspective, reference values derived primarily from lowland populations may not always be directly applicable to high-altitude residents. Establishing blood pressure reference values based on large-scale datasets from high-altitude populations themselves would therefore be an important objective for future research.

We next address the classification of polycythemia based on hemoglobin concentration. In our article, polycythemia in women was defined as a hemoglobin concentration ≥ 16 g/dL, based on previous studies that categorized “moderate polycythemia” (men: 18–21 g/dL; women: 16–19 g/dL) and “excessive polycythemia” (men: ≥ 21 g/dL; women: ≥ 19 g/dL) [3, 4]. In our analysis, moderate polycythemia or higher was broadly grouped under the term polycythemia. Craig et al. noted that studies of high-altitude populations often adopt the concept of “excess erythrocytosis,” using a threshold of hemoglobin ≥ 19 g/dL for women [5]. This definition is based on an international consensus statement issued by the International Society for Mountain Medicine (ISMM), which integrates current knowledge on chronic and subacute high-altitude diseases. We agree that the use of internationally recognized criteria facilitates comparability across studies. We also acknowledge that the criteria used in our study included relatively mild elevations in hemoglobin concentration, which may encompass levels of limited clinical significance.

At the same time, the direct applicability of any single criterion warrants careful consideration in populations such as Tibetan highlanders, who exhibit genetic adaptations to hypobaric hypoxia. The ISMM consensus itself recognizes that geographic and population-specific factors influence the development of chronic mountain sickness and related conditions. Tibetan highlanders are characterized by physiological adaptations including enhanced vasodilation and relatively modest increases in hemoglobin concentration. As a result, the physiological implications of a given hemoglobin level may differ between Tibetan and Andean highlanders.

In our study, the rationale for including mild elevations in hemoglobin concentration was not sufficiently articulated in the original article, and we acknowledge this limitation. We agree that establishing clinically meaningful reference values requires health data from very large populations. In this context, the term “moderate erythrocytosis” may indeed be more appropriate than “polycythemia.” The conceptual framework and thresholds for erythrocytosis in high-altitude populations remain subjects of ongoing discussion, and further research is needed to establish population- and ethnicity-specific reference values.

Additional health examinations conducted in Tsarang in 2025 indicated that 6.34% of women had hemoglobin concentrations exceeding 16 g/dL. These measurements were obtained from venous blood samples analyzed locally, rather than from transcutaneous measurements, and are therefore considered reliable. Among men, 2% had hemoglobin concentrations exceeding 19 g/dL, and 1% exceeded 21 g/dL. Thus, residents meeting criteria corresponding to moderate erythrocytosis were observed in our surveys. While the data presented in the original article were derived from a 2017 survey, the 2025 survey included a higher proportion of younger participants. This demographic difference may partly explain the lower prevalence of moderate erythrocytosis observed in 2025.

**Fig. 1:**
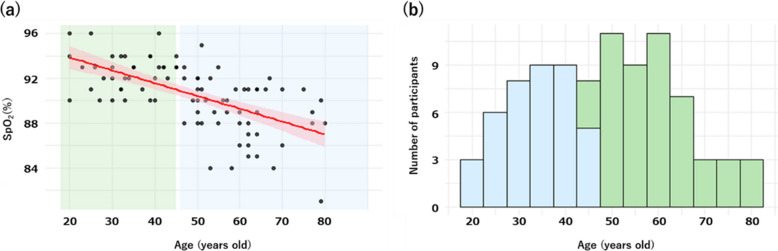
Relationship between participants’ SpO₂ and age and age group classification. **a** Scatter plot illustrating the relationship between age and SpO₂ among female participants in the study by Arima et al., with fitted regression lines ± standard error. The green region represents participants aged ≤ 45 years, and the blue region represents those aged ≥ 46 years. **b** Histogram illustrating the age distribution of female participants. The green bars represent those aged ≤ 45 years, and the blue bars represent those aged ≥ 46 years

Regarding oxygen saturation, when a cutoff value of 90% was applied, 28% of women in our dataset fell below this threshold, compared with 67% in the dataset reported by Craig et al. In our data, SpO₂ was negatively correlated with age (Fig. 1-(a)), which is consistent with the higher proportion of individuals with SpO₂ < 90% in the older population studied by Craig et al. We agree that environmental hypoxia and pathological hypoxia cannot be distinguished solely on the basis of SpO₂ measurements. We also recognize that our use of the term “hypoxemia” and the application of a 90% cutoff value may have caused confusion. In our study, this cutoff was intended as a descriptive classification rather than as an indicator of pathology, and terminology such as “low oxygen saturation group” would likely have been more appropriate.

At present, the relationship between oxygen saturation and tissue- or brain-level hypoxia under high-altitude conditions has not been clearly established, and no universally accepted definition of hypoxemia exists for high-altitude populations. We therefore agree that caution is warranted when applying the term “hypoxia” in this context. We do not interpret chronically low oxygen saturation among Tsarang residents as indicative of adverse health outcomes. As with hemoglobin concentration, the clinical significance of SpO₂ and the establishment of appropriate reference values remain open questions, and future studies will be essential for developing ethnicity-specific criteria. In our article, we cited World Health Organization reference values for completeness [6].

### SNP selection and the impact of differences in study populations on disease prevalence

In this study, we genotyped a single-nucleotide polymorphism (SNP) in EGLN1 among residents of Tsarang to examine whether this SNP—previously reported to be associated with susceptibility to acute hypobaric hypoxia in Asian lowland populations and to occur at high frequency in high-altitude populations—was associated with oxygen circulation dynamics in this setting [7]. In response to the comments regarding our SNP selection, we provide additional clarification of the rationale underlying this choice.

As discussed above, we recognize the importance of carefully justifying the selection of reference values for each physiological condition. At the same time, differences in participant characteristics between the two studies likely contributed to discrepancies that cannot be attributed solely to reference values. Our study included participants aged 18 years and older, whereas the study by Craig et al. focused on women aged 46 years and older. This substantial difference in age distribution is likely to have influenced the observed health profiles.

The age distribution of participants in our study is shown in Fig. 1-(b). Even when analyses are restricted to subsets matched by sex, age, and marital status, findings derived from a study including a broad adult age range may not be directly comparable to those from a study restricted to older individuals. Accordingly, comparisons between the two studies should be interpreted with careful consideration of differences in study populations and analytical conditions.

## Conclusions

In light of the thoughtful comments provided by Sienna R. Craig and colleagues, we have reaffirmed the importance of critically evaluating the reference values and measurement approaches used in epidemiological studies of high-altitude populations. The issues raised in their commentary, together with those discussed here, provide valuable perspectives on how population health in high-altitude settings can be conceptualized and assessed. We are grateful for the opportunity to engage in this scholarly exchange and sincerely appreciate the contribution of Professor Beall and colleagues in fostering constructive discussion in the field of high-altitude research.

## Data Availability

No datasets were generated or analysed during the current study.

## Abbreviations

BMI: Body mass index
ISMM: International Society for Mountain Medicine
SNP: Single-nucleotide polymorphism
